# Low-cost desktop learning factory to support the teaching of artificial intelligence

**DOI:** 10.1016/j.ohx.2024.e00528

**Published:** 2024-04-19

**Authors:** Eduardo Orozco, Paulo C. Cárdenas, Jesús A. López, Cinthia K. Rodriguez

**Affiliations:** a Department of Automation and Electronics, Universidad Autónoma de Occidente, Cali, Colombia; b Department of Physics and Mathematics, Universidad Autónoma de Manizales, Manizales, Colombia

**Keywords:** Machine learning, Artificial intelligence, Education k-12, Teaching strategy

## Abstract

The following document details low-cost hardware and open-source available software tools that can be combined to support active teaching methodologies like Problem-Based Learning (PBL) and incorporate work-oriented technological skills in students. This proposal presents a prototype of Open Educational Resources (OER) that integrates software and hardware tools for the specific purpose of facilitating instruction in Artificial Intelligence. The hardware consists of affordable electronic devices, including an Arduino board, servo motors, sensors, a relay and a motor, all integrated into a scaled conveyor belt. On the other hand, open software was used to implement an image classification program with different features (shape, color, size, among others). The exact construction steps, circuits, and code are presented in detail and should encourage other scientists to replicate the experimental setup, especially if they are looking for experimental teaching of artificial intelligence, since the system allows object classification using the machine learning paradigm to facilitate the teaching of artificial intelligence concepts with computer vision concepts.

## Graphical abstract

**Figure:**
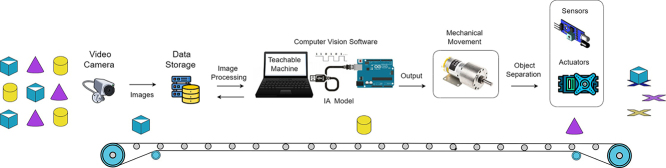

## Specifications table

**Table:** 

Hardware name	Low-Cost Prototype to Support the Teaching of Artificial Intelligence.

Subject area	•Engineering and Material Science • Educational Tools and Open Source Alternatives to Existing Infrastructure • Industrial Simulation

Hardware type	•Artificial Intelligence • Automated control system based on artificial vision • Electrical engineering and computer science • Mechanical engineering and materials science

Closest commercial analog	JETSON NANO robotic arm with artificial vision based on AI for image classification.

Open source license	GNU General Public License (GPL) 3.0

Cost of hardware	US$ 850.28

Source file repository	http://doi.org/10.17605/OSF.IO/4BDKU

## 1 Hardware in context

Currently, active teaching methods are widely used and studied across various academic fields and levels of education. Research suggests that STEM-based active teaching practices promote effective development of student knowledge [1]. In STEM courses, students typically utilize and modify contemporary technologies in diverse learning settings [2], [3], [4]. It is worth noting that students do not necessarily require cutting-edge or expensive technology to integrate technological processes and concepts [5]. This feature is particularly significant for developing countries and technological democratization [6].

Our focus is on highlighting various technologies for teaching that utilizes automated servomotors with image classification on conveyor belts to classify objects, encompassing the concept of Industry 4.0 in the educational context. To contextualize our prototype, we categorize the related literature into three distinct categories: servomotors, conveyor belts, and computer vision. References to servomotors are primarily related to embedded systems used in robotics. For instance, prototypes are used for developing industrial robots capable of detecting objects in motion on a conveyor belt, such as those mentioned in [7]. Additionally, [8] includes a simulation of a robotic arm picking objects from a static or dynamic position on a conveyor belt. For additional educational purposes, refer to [9], where the authors use open software and hardware to teach robotics, data acquisition, and related concepts. Similarly, in [10], a camera is added to a robot to identify and utilize pertinent information from the surroundings to execute a task. Computer vision has numerous applications, including determining fruit quality as examined in [11], product quality, and even garbage classification as stated in [12], among others.

General-purpose systems that can be adapted to the aforementioned tasks have already been developed. For instance, the LEGO MINDSTORMS EV3 set enables the use of a conveyor, similar to those in industries, for moving a ball through a path using a motor and a sensor [13]. This system can also be configured to activate a sorting machine. Similarly, fischertechnik offers a comparable industrial simulation for transporting and selecting objects by color, see [14]. Even more advanced systems have been created, as exemplified by the JetMax robotic arm. This device incorporates an HD camera and a Jetson Nano NVIDIA chip, which enables its use in AI applications. Its capabilities include object identification and color sorting, object tracking, shape classification, industrial process simulation, and other functions [15].

The purpose of the prototype is to offer an affordable educational instrument that simulates transportation and object classification by using electro-mechanical and automated components. The object classification algorithm is based on machine learning training techniques and is controlled by the Arduino UNO. Operating the system is straightforward; thus, the mathematical aspects connected to the algorithms and programming are inconsequential. Consequently, the prototype can be included in secondary STEM courses to inspire students to pursue engineering or development professions. Additionally, the objective is to create affordable prototypes that enable educators in developing countries to incorporate emergent technologies for school students.

This prototype provides students with a physical and tangible platform to interact with abstract artificial intelligence concepts. The use of lightweight and inexpensive materials, such as Medium Density Fiberboard (MDF), makes the device accessible and easy to transport, allowing it to be used in a variety of educational settings. Furthermore, the integration of hardware components such as the Arduino Uno development board, infrared sensors, and actuators like servo and gear motors, enables students to conduct hands-on experiments on the interplay between hardware and software in practical scenarios. This not only enhances understanding of the theoretical principles of artificial intelligence, but also fosters critical thinking and problem solving, fundamental skills in STEM (science, technology, engineering and mathematics) learning.

## 2 Hardware description

**Fig. 1:**
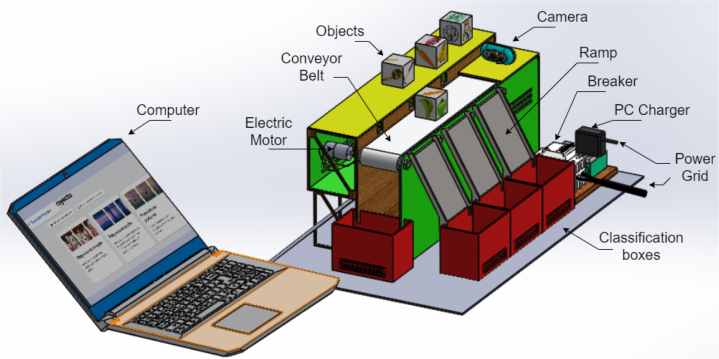
General scheme of the prototype.

**Fig. 2:**
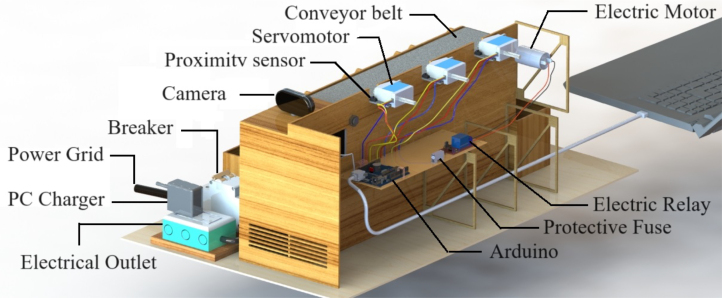
General scheme of the prototype: Back view. Main parts are presented.

The prototype (Desktop Learning Factory) works by placing an object in front of the camera and activating the image recognition function, which utilizes a previously implemented classification algorithm. This recognition algorithm assigns a box according to the detected characteristics and activates the driving conveyor belt motor once the system identifies the class to which the object belongs. This recognition algorithm assigns a box according to the detected characteristics and activates the driving band motor once the system identifies the class to which the object belongs. When the object passes its corresponding checkbox, a proximity sensor detects it, and this causes the belt to be stopped and a servomotor to be activated, which pushes the object towards the box. In Fig. 1, Fig. 2 it is presented the general idea of the prototype.

To develop the classification model, the free software “Teachable Machine” was used. This tool facilitates the acquisition of images by the camera and their subsequent classification into classes. Once the classes have been defined, which can be from 2 to 4 in this case, the program is fed with the previously classified images. Consequently, the software produces a model that facilitates the classification of images.

To set up the properties of the object, a set of images of the product in question is captured and stored. These images are then analyzed by an artificial intelligence (AI) algorithm that can identify the essential characteristics required for the classification process. The model is then trained and transferred to a development board. Then, a control software initiates a process that activates servomotors to enable mechanical movement for object classification based on detected features. It is important to note that the design can possess three distinct characteristics, in addition to one for objects that cannot be classified.

The system consist in the following coupled subsystems: electro-mechanical, optical and electronic hardware that all together support the operation.

### 2.1 Electro-mechanical hardware

**Fig. 3:**
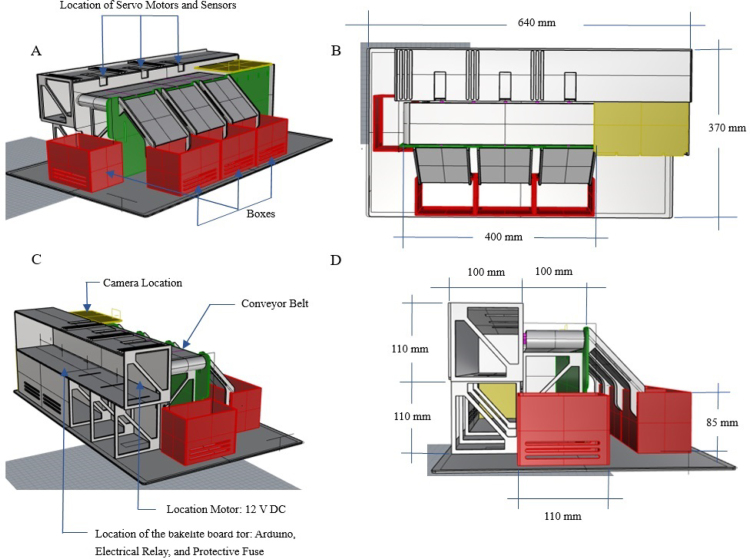
Structure of the prototype. (A) Location of servo motors, sensors and boxes. (B) Top view dimensions. (C) Location of camera, conveyor belt, motor and PCB board. (D) Side view dimensions. (For interpretation of the references to color in this figure legend, the reader is referred to the web version of this article.)

A mechanical structure was design accordingly to two main restrictions: low cost materials and portability such that the dimensions of the system fit usual classroom desktops. In Fig. 3 (colors are for reference) the structure is presented, it shows the conveyor belt of dimensions: 400 mm × 80 mm × 31 mm, object ramps with dimensions: 88 mm × 71.3 mm × 148.12 mm and classification boxes with dimensions: 110 mm × 75 mm × 85 mm. The base area of the system is: 499 mm × 93 mm × 223 mm, inside we put the Arduino card, servomotors, relay and fuse. The whole structure was made of 3 mm thick medium density fibreboard (MDF). Top and lateral view of the structure are presented in Fig. 3(B) y (D). The whole structure has dimensions: 640 mm × 370 mm × 220 mm. The conveyor belt is made of thermoplastic polyurethane TPU material and four aluminum support rollers measuring 85 mm by 26 mm were utilized. This is powered by a 25D × 54L mm metal gear motor operating at 12VDC, controlled by the Arduino board using a 5-V SRD-5VDC-SL-C relay. A Micro Servo Motor SG90 triggers an arm, which executes an simple motion that moves objects in accordance with the software control and object’s predefined feature. The servomotors’ position is displayed in Fig. 3(A).

### 2.2 Optical hardware

Optical hardware comprises a full HD (1080p) webcam connected to a computer via a USB port with plug and play functionality, as well as an infrared sensor (Note that the webcam can be freely chosen). The hardware serves two primary purposes in the system function. Firstly, the webcam is used to capture the object images that are called product images, those images are stored and then they feed the ML training model. Secondly, once the object enters the belt, the webcam is activated, because the object is detected for the infrared sensor that communicates with the computer and the control software.

### 2.3 Electronic hardware

The main control system is operated by an infrared sensor and ARDUINO ONE card. The infrared sensor is positioned next to the servomotors, when the object passes by the sensor the belt stops for a short amount of time while the camera operating as an optic sensor takes the image and contrasts it with the training AI model to make a decision related to the motion of the servomotor. The sensor is composed by an emission infrared LED, and a BPV10NF photodiode that senses the reflected light from the object that the acts as an object detection.

**Fig. 4:**
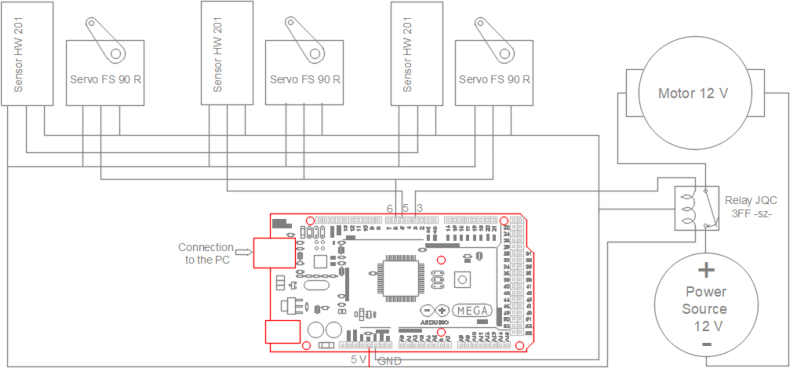
Connection diagram.

**Fig. 5:**
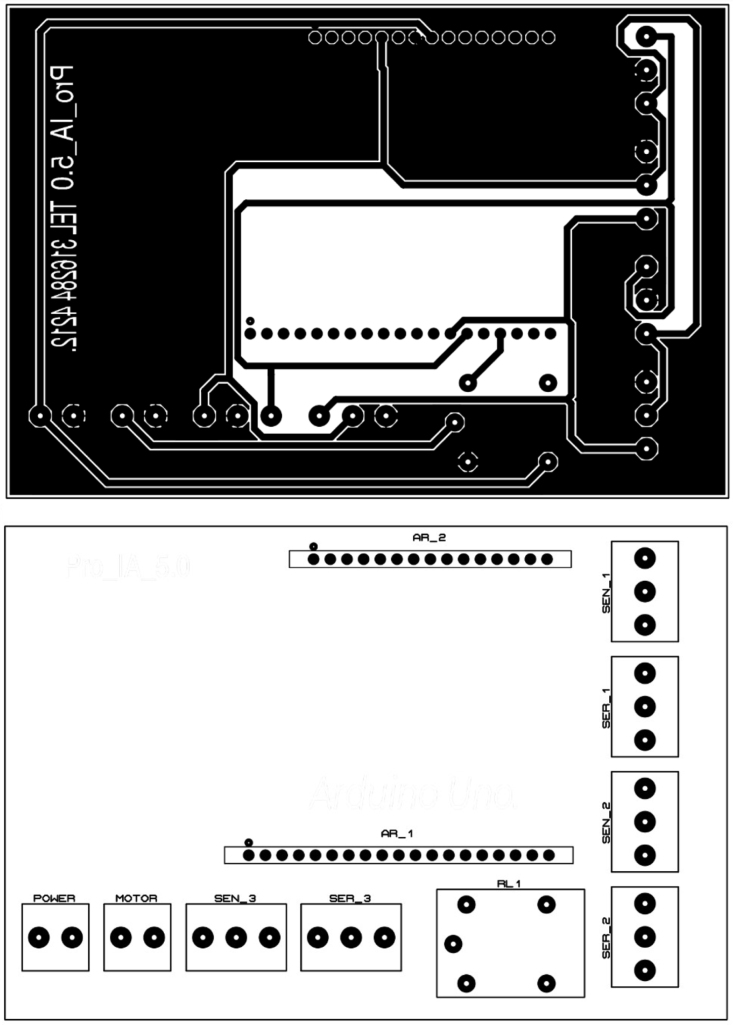
PCB design.

On the other hand, a 250 mm × 90 mm PCB was designed and printed (see Fig. 5), to organize the connection of the elements such as servomotors, sensors and electric motor with 20 cm connection cables serving as a conduit. The connection of these elements to the Arduino One board is displayed in Fig. 4.

In this approach, technological support is achieved through a device that consists of both hardware and software components, seamlessly integrated for maximum efficiency. With this device, students can fully immerse themselves in computer vision concepts within the realm of artificial intelligence (AI). The device not only guarantees an enhanced learning experience but also widens the scope of skills, specifically focused on participating in activities related to cutting-edge technologies such as machine learning.

Participation in AI-related activities enables students to enhance their expertise in computer vision and develop a deeper interest in engineering methods that can be utilized to address problems related to their requirements. Additionally, for user convenience, it is preferable that the device system reduces the necessity for complicated mathematical computations or the creation of intricate programming languages with complex code. Therefore, this device will be of interest to those looking for:

• To learn about a tool that supports the teaching of artificial intelligence concepts.
• Recreate a practical scenario of object classification from images.
• Replicate an industrial process at scale.

### 2.4 Software description

***Teachable Machine***

Teachable Machine’s software architecture is based on an intuitive user interface, an easy-to-use machine learning engine, and a hardware abstraction layer that allows users to create and train machine learning models without deep programming or data science skills. In this case, this software is used to create the image classification model that will be used to classify the objects on the conveyor belt (the process of creating the model is explained in Section 6).

***Arduino IDE***

**Table 1:** Additional material cost.

Component	Description	Qty	Unit cost (US$)	Total cost (US$)
3 mm mdf wood structure	60 × 40 × 20 (cm)	1	33.73	33.73
Wood screws	1 cm × 1/16${}^{\prime \prime }$	24	0.011	0.25
Wood glue	200 g per jar	1	1.05	1.05
Welds all	40 g per box	1	2.74	2.74
Aluminum rollers	8 cm Length × 2 cm Diameter	4	4.22	16.87
Ball type bearings	Ref. 627-ZZ	4	0.74	2.95
Millimetric bolts with nuts, and washers	12 cm × 5/16${}^{\prime \prime }$	4	0.74	2.95
Polyethylene band	7,5 cm × 80 cm	1	6.32	6.32
Support for servo motors Resin, with pin and rack	06B 10T 10 teeth pitch/45 teeth pitch	3	6.32	18.97

**Total cost of support materials (US$)**				**85**.**84**

In this IDE it is possible to write programs to control the behavior of the devices connected to the Arduino board. These programs, known as “sketches”, are uploaded to the board via a USB cable, which facilitates programming and debugging.

This Arduino code configures four servo motors, four LED sensors and a relay. In addition, it uses a loop() to read from the serial port and control the servomotors based on the commands received.

1. Initial configuration: The Servo.h library is included to manage the servo motors. Several pins are defined for the servomotors (myservo, myservo1, myservo2), and sensors (sensorPin, sensorPin1, sensorPin2). A pin is also defined for the relay to control the electric motor that moves the conveyor belt.
2. “setup”():
  • Serial communication starts at 9600 baud (“Serial.begin(9600)”).
  • The servomotors are set to the corresponding pins and their initial position (“90” degrees) is established.
  • The pins of the LED sensors and the relay are configured as outputs (“OUTPUT”).
3. “loop()”:
  • Reads a character from the serial port and stores it in “result”.
  • Within a “while” loop, it checks if data is available on the serial port.
  • Depending on the value of “result”, one of the following code blocks is executed:
    – A: It reads the sensor “sensorPin”. If it detects an obstacle, it waits 10 000 ms to stop the belt and moves “myservo” to push the object through the rack rail.
    – B: Similar to “A” but for “sensorPin1” and “myservo1”.
    – C: Similar to “A” but for “sensorPin2” and “myservo2”.
    – D: It reads “sensorPin” and if it detects an obstacle, it sends the signal “result” so as not to stop the movement and the object reaches box 4 and then waits five seconds to stop the conveyor belt.
  • Finally, wait one second before restarting the loop.

***P5.js***

P5.js is a JavaScript library that enables the creation of interactive graphics and visualizations on the web. While P5.js is not intended for interfacing with external hardware like Arduino, it can be used with additional libraries such as p5.serialport.

P5.serialport is used in P5.js to establish bidirectional communication between the P5.js sketch and the Arduino program through the serial port. This enables the sending of data from the P5.js sketch to Arduino to control devices or read sensors, as well as receiving data from Arduino to display or process it in the P5.js sketch.

P5.serialport is utilized in P5.js to interface with the Arduino because it offers a convenient method to establish bidirectional serial communication between a JavaScript program (P5.js) and a microcontroller like the Arduino. This enables the creation of interactive and innovative projects that combine software and hardware.

## 3 Design files

***Design files summary***

The best way to determine the proper use of each design file is to refer to the descriptions provided in Section 5. The BOM below lists all the CAD files made.

**Table:** 

Design filename	File type	Open source license	Location of the file
Base.SLDPRT	CAD	GNU GPL v3	osf.io/4bdku

Base-Breaker.SLDPRT	CAD	GNU GPL v3	osf.io/4bdku

Checkbox.SLDPRT	CAD	GNU GPL v3	osf.io/4bdku

Ramp.SLDPRT	CAD	GNU GPL v3	osf.io/4bdku

Ramp-Sides.SLDPRT	CAD	GNU GPL v3	osf.io/4bdku

ConveyorBeltSupports-Front.SLDPRT	CAD	GNU GPL v3	osf.io/4bdku

ConveyorBeltSupports-Back.SLDPRT	CAD	GNU GPL v3	osf.io/4bdku

Structure-Support.SLDPRT	CAD	GNU GPL v3	osf.io/4bdku

Structure-LevelSeparator.SLDPRT	CAD	GNU GPL v3	osf.io/4bdku

Structure-BackCover.SLDPRT	CAD	GNU GPL v3	osf.io/4bdku

Structure-SideCoverL1.SLDPRT	CAD	GNU GPL v3	osf.io/4bdku

Structure-SideCoverL2.SLDPRT	CAD	GNU GPL v3	osf.io/4bdku

Structure-SupportCoverC.SLDPRT	CAD	GNU GPL v3	osf.io/4bdku

Structure-TopCover.SLDPRT	CAD	GNU GPL v3	osf.io/4bdku

ServomotorRack.stl	STL	GNU GPL v3	osf.io/4bdku

ServomotorPinion.stl	STL	GNU GPL v3	osf.io/4bdku

Rollers.SLDPRT	CAD	GNU GPL v3	osf.io/4bdku

PCBDesign.pdf	PDF	GNU GPL v3	osf.io/4bdku

			

## 4 Bill of materials summary

The cost of each of the elements, devices, and parts used in the prototype is detailed. Software tools are freely available. This section has been divided into two budgets (see Table 1, Table 2). Also, the equipment used are detailed in Table 3

**Table 2:** Cost of electronic elements and devices.

Part	Description	Qty	Unit cost (US$)	Total cost (US$)
Arduino development board	Arduino Uno	1	9.49	9.49
USB cable for Arduino	Type A - B × 1 m	1	6.54	6.54
Protoboard small	4.8 × 3.5 × . 9 cm	2	1.69	1.69
Relay 5 V DC	SRD-5VDC-SL-C	1	0.95	0.95
Motor - gearbox Pelv motor	4 kg 155 Rpm 12 V	1	7.38	7.38
Adapter (Power supply)	Variable voltage 1.5 V A 12 V	1	10.54	10.54
Micro servo motor sg90	SG90 180 Degrees 9G	3	2.32	6.96
Infrared obstacle avoidance sensor	3-pin switch	3	7.00	21.00
HD digital camera	480P/720P/1080P	1	9.49	9.49
Electrical extension power cable	2 m	1	6.75	6.75
Jumper cables for test board	10 cm M-m - Paq × 40 pz	1	2.53	2.53

**Cost of electronic elements and devices (US$)**				**84**.**98**

**Table 3:** Cost of equipment required.

Component	Description	Qty	Unit cost (US$)	Total cost (US$)
Computer	Simple laptop	1	527.04	527.04
Digital uni-t multimeter	Ut33c+ Temperature - Ac/dc	1	13.70	13.70
Dewalt variable speed drill	1/2 710 W 2.800 Rpm	1	72.94	72.94
Professional metallic scalpel	6${}^{\prime \prime }$ Reinforced - Truper	1	44.000	9.28
Stanley open-end/fixed wrench set	8 pcs. Metric 6 ∗ 7-20 ∗ 22	1	24.67	24.67
Stanley basic screwdriver set	10 Pzs. 1/4 × 4${}^{\prime \prime }$	1	12.65	12.65
DeWalt titanium drill bit set	8.75 × 8.75 × 1.38 in	1	13.70	13.70
Flexometer 3mt Lufkin	10.2 cm × 9.7 cm -L510ME	1	1.48	1.48
Steel square ruler redline	Fixed 10Pg 250Mn	1	4.01	4.01

**Total equipment cost (US$)**				**679**.**46**

## 5 Build instructions

**Fig. 6:**
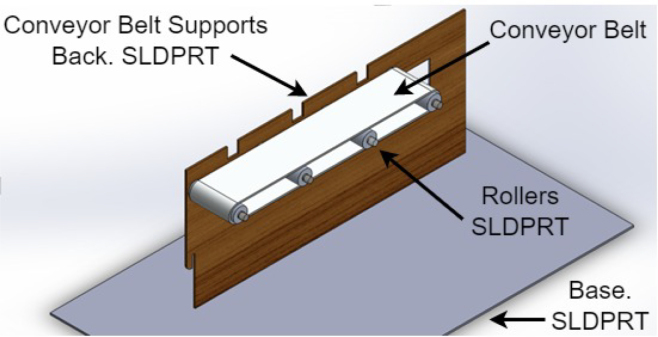
Step 2 - Build instructions.

**Fig. 7:**
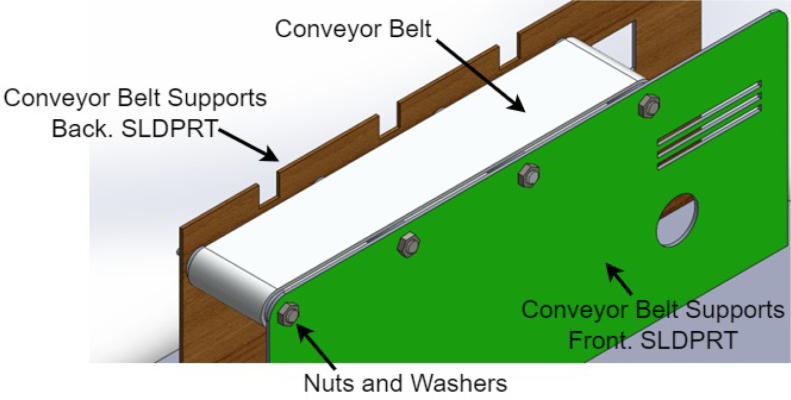
Step 3.1 - Build instructions.

**Fig. 8:**
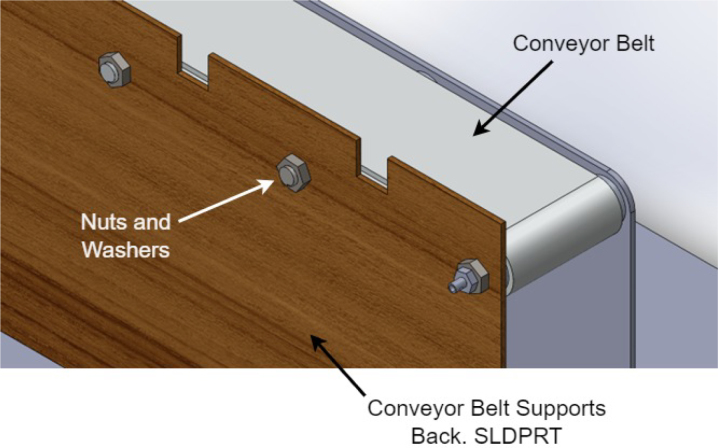
Step 3.2 - Build instructions.

**Fig. 9:**
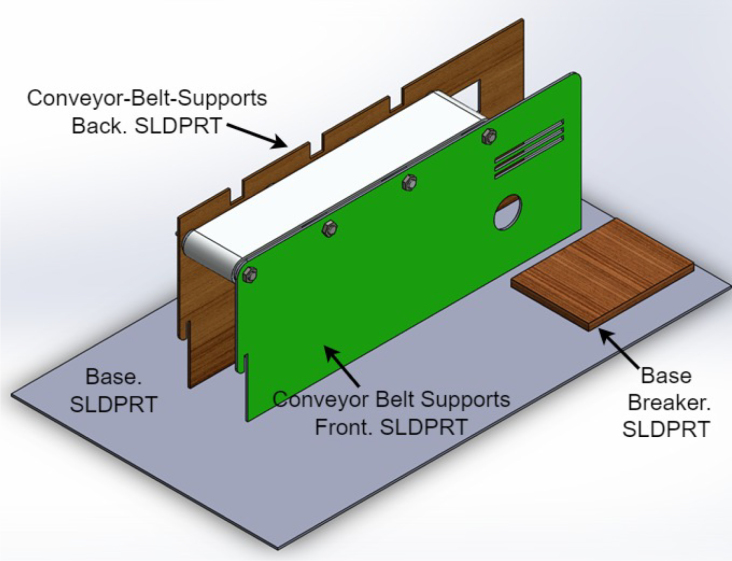
Step 4 - Build instructions.

**Fig. 10:**
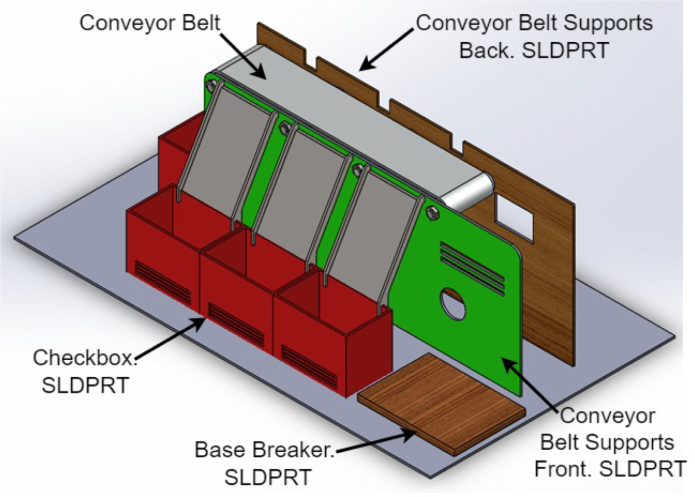
Step 5 - Build instructions.

**Fig. 11:**
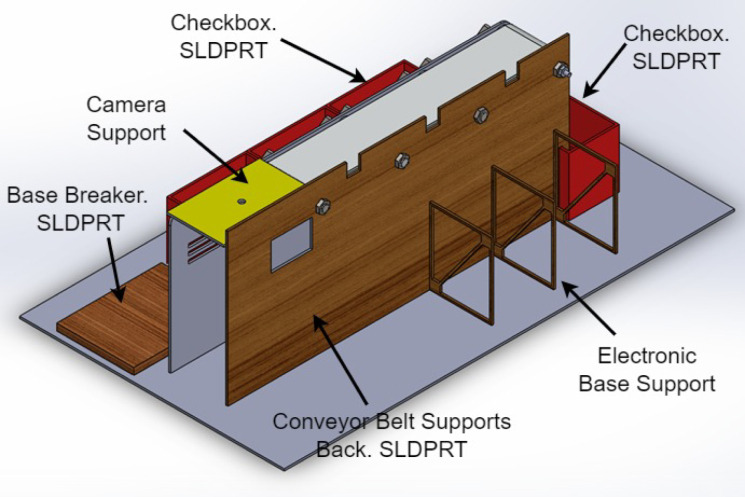
Step 6 - Build instructions.

**Fig. 12:**
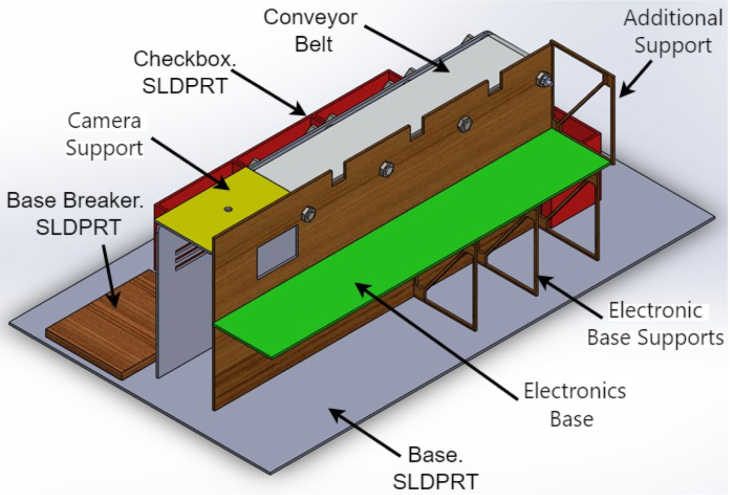
Step 7 - Build instructions.

**Fig. 13:**
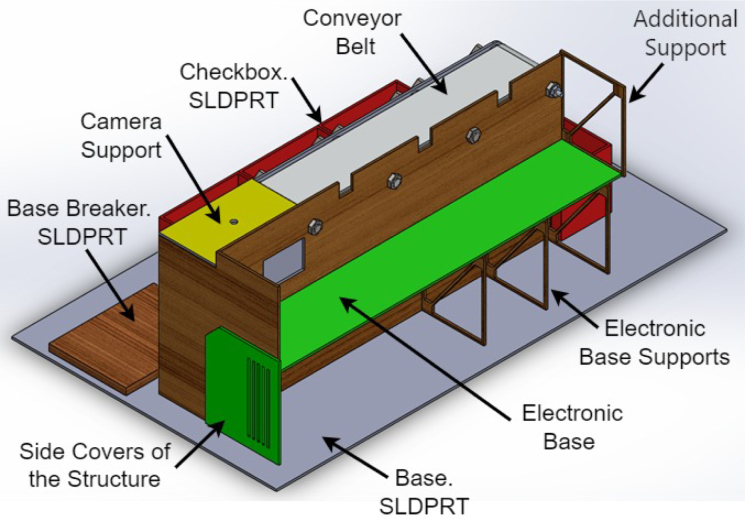
Step 8 - Build instructions.

**Fig. 14:**
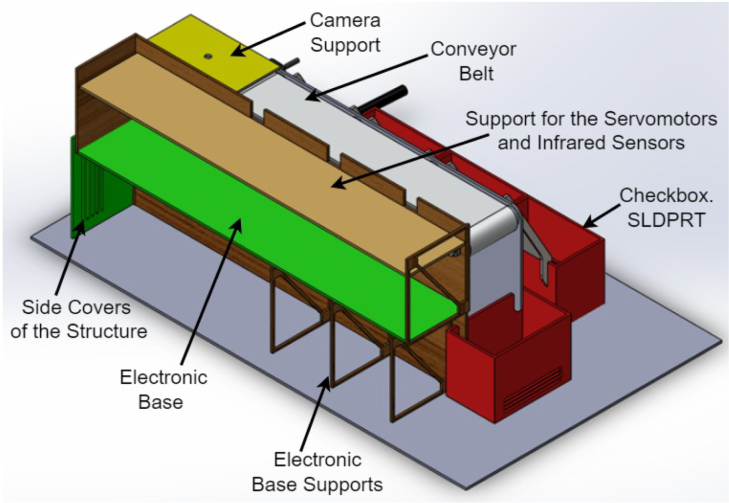
Step 9 - Build instructions.

**Fig. 15:**
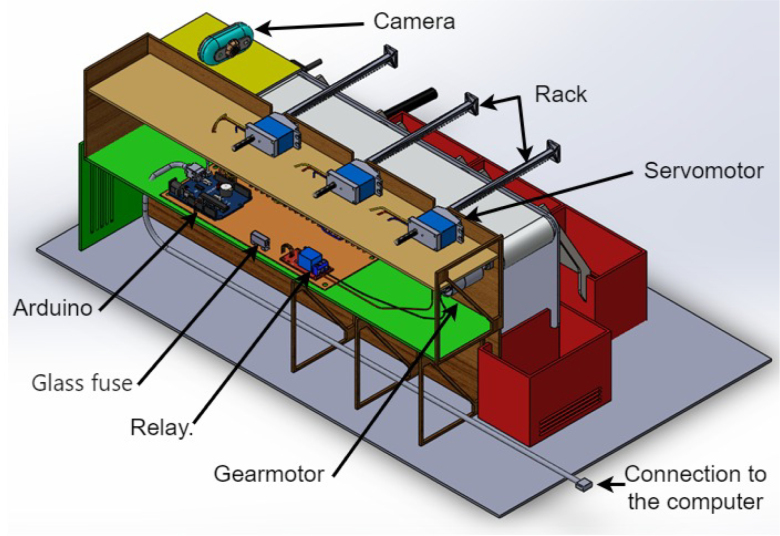
Step 11 - Build instructions.

**Fig. 16:**
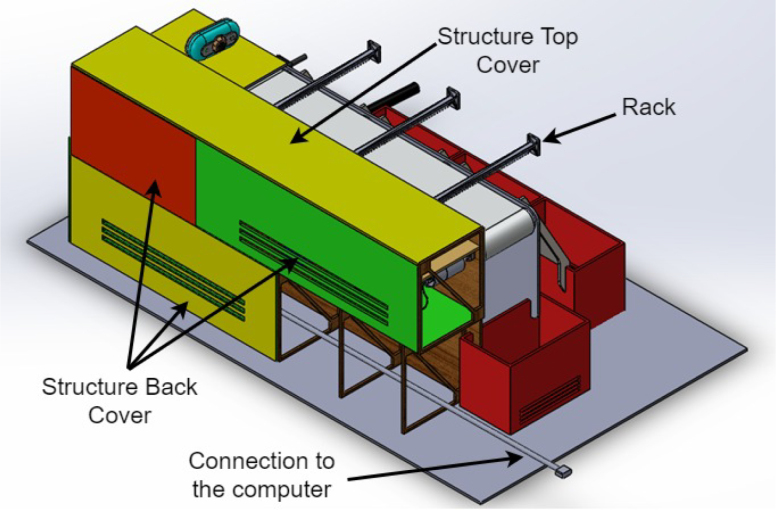
Step 12 - Build instructions.

Listed below are the steps to follow for build instructions:

1. The ‘.SLDPRT’ files (available in Section 3, titled ‘Design files’) that make up the pieces of the structure were accommodated and cut in MDF with a laser machine. These pieces were designed to interlock like a puzzle for easy assembly. Furthermore, it should be noted that to ensure the robustness and stability of the assembly, wooden supports, screws, and glue were utilized during the construction of the structure.
2. The base (Base.SLDPRT) served as the starting point for the conveyor belt’s back support, which was placed vertically using the ConveyorBeltSupport-Back.SLDPRT file. Four supporting aluminum rollers measuring 85 mm by 26 mm (Rollers.SLDPRT) were then installed. The conveyor belt, made of thermoplastic polyurethane (TPU) and vulcanized to a length of 400 mm by 80 mm wide, was placed on these rollers (see Fig. 6).
3. Subsequently, the conveyor belt’s front support was installed and secured using four nuts and their corresponding washers, as illustrated in Fig. 7. Furthermore, the appropriate modification was made on the back support, including the placement of the coupling to position the motor, as shown in Fig. 8.
4. The base of the breaker, which corresponds to the Base-Breaker.SLDPRT file, was adhered to the structure’s base (see Fig. 9).
5. Using industrial glue, the boxes that correspond to the Checkbox.SLDPRT file, designed to contain the classified cubes, were assembled and the ramps designed to facilitate the descent of these cubes (derived from the Ramp.SLCPRT and Ramp-Sides.SLDPRT files) were assembled (see Fig. 10).
6. Next, the camera support (Structure-SupportCoverC.SLDPRT file) was installed between the conveyor belt supports. Additionally, the three first level supports were attached to the back of the structure (Structure-Support.SLDPRT file) as shown in Fig. 11.
7. The electronics base, taken from the Structure-LevelSeparator.SLDPRT file, is placed on top of the level supports, and an additional support is added to reinforce the structure cover (Structure-Support.SLDPRT file), as shown in Fig. 12.
8. To secure a stable support for the electronic devices, the side covers of the structure are placed. These covers correspond to the files Structure-SideCoverL1.SLDPRT and Structure-SideCoverL2.SLDPRT, with the latter being removable, as shown in Fig. 13.
9. The support for the servomotors and infrared sensors, identified in the Structure-LevelSeparator.SLDPRT file, is now positioned (see Fig. 14).
10. The printed circuit was fabricated on a PCB board according to the design shown in Fig. 5. The necessary soldering was done on this board to integrate the Arduino Uno and the relay. Then, the connection of the motor, the servomotors and the infrared sensors was made using wires following the diagram shown in Fig. 4.
11. The entire electronic section, including the previously mentioned printed circuit and all of its connected components, the camera, voltage adapter, and current protection breaker, was subsequently installed in the structure. Additionally, a 3D-printed rack and pinion was integrated with each servomotor to assist in pushing the sorted cubes (see Fig. 15).
12. To complete this section of the design, the back of the structure is covered with the part corresponding to the Structure-BackCover.SLDPRT file (see Fig. 16).

**Fig. 17:**
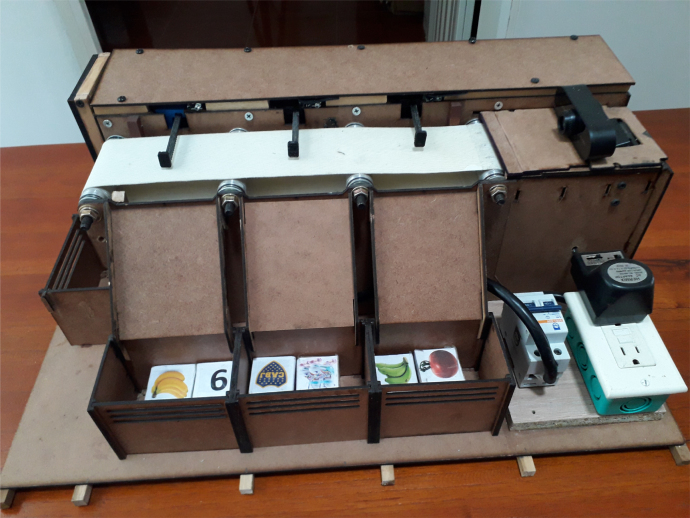
Final prototype.

The physical device is presented visually in the Fig. 17.

## 6 Operation instructions

**Fig. 18:**
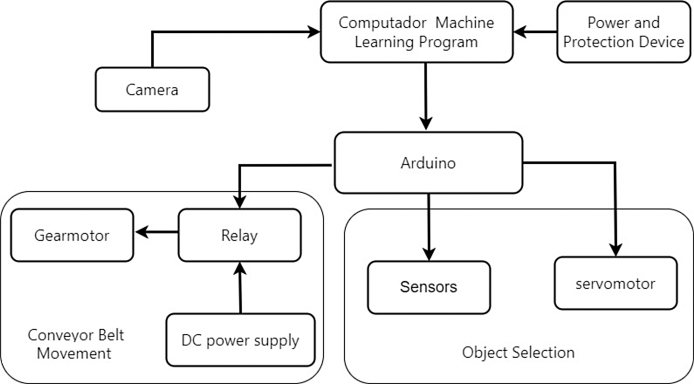
Prototype connection.

**Fig. 19:**
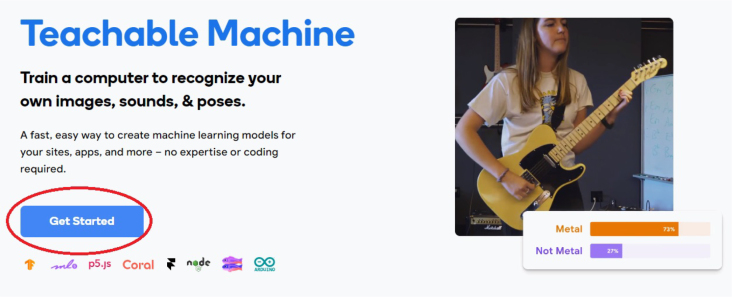
Step 6 - Operating instructions.

**Fig. 20:**
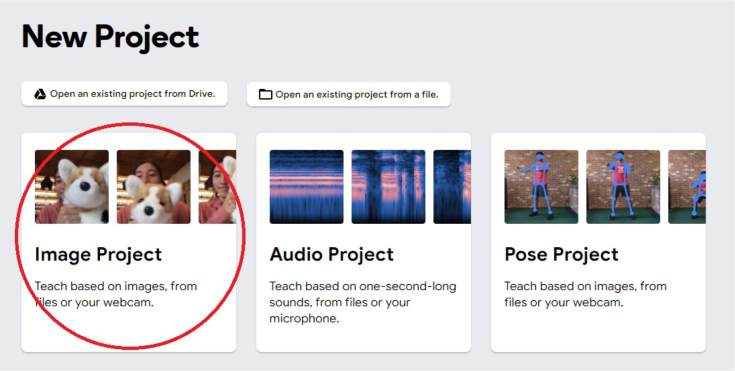
Step 7 - Operating instructions.

**Fig. 21:**
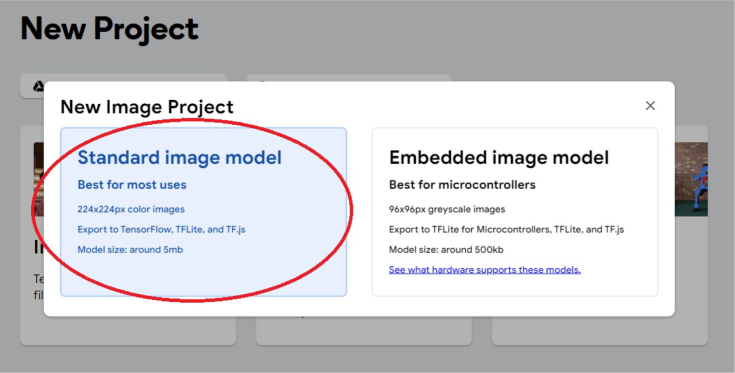
Step 8 - Operating instructions.

**Fig. 22:**
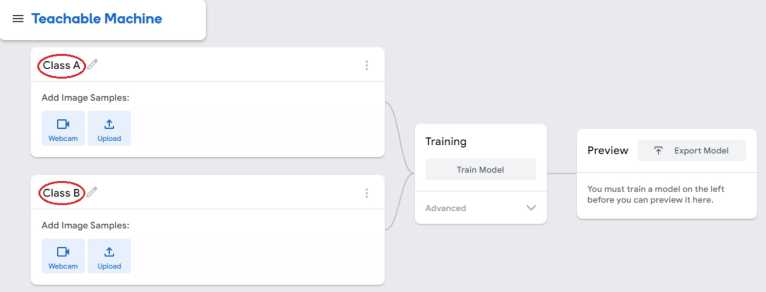
Step 9 - Operating instructions.

**Fig. 23:**
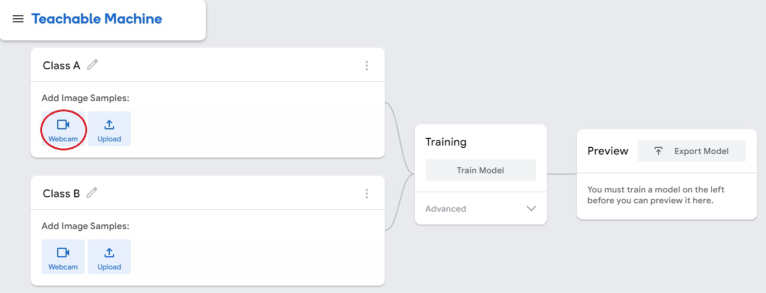
Step 10 - Operating instructions.

**Fig. 24:**
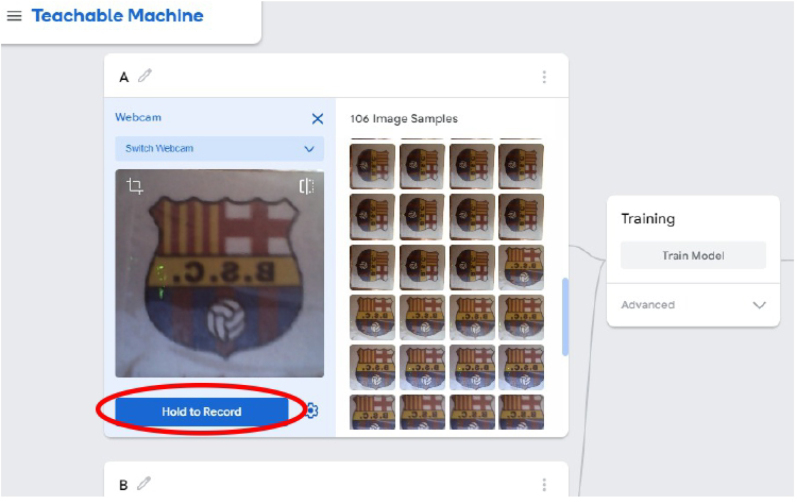
Step 11 - Operating instructions.

**Fig. 25:**
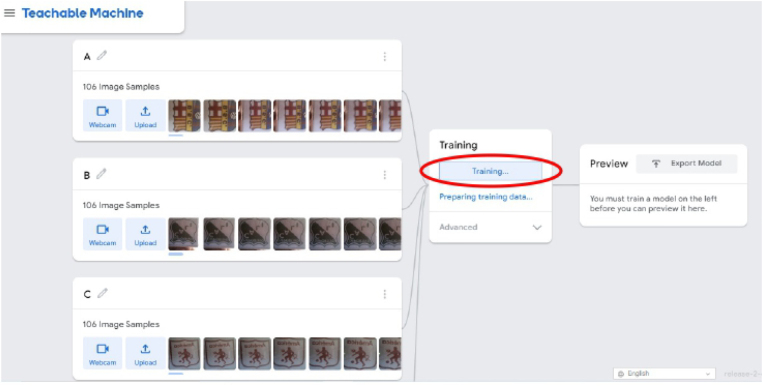
Step 12 - Operating instructions.

**Fig. 26:**
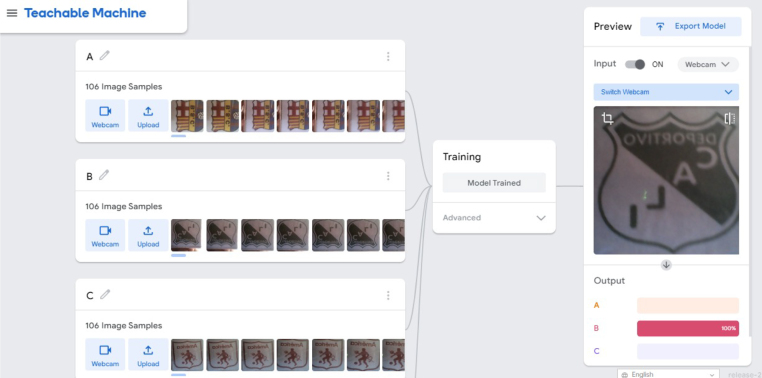
Step 13 - Operating instructions.

**Fig. 27:**
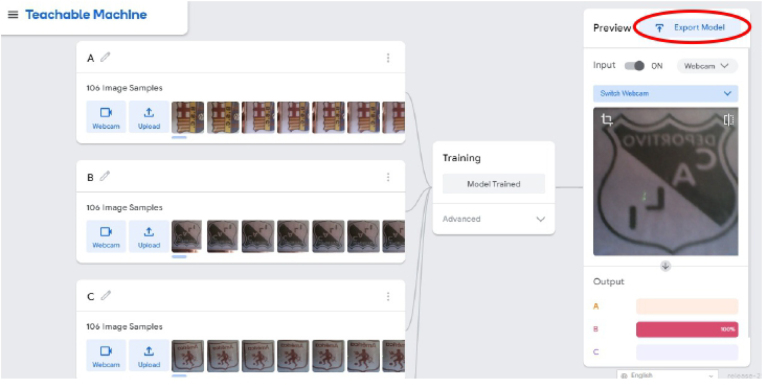
Step 14 - Operating instructions.

**Fig. 28:**
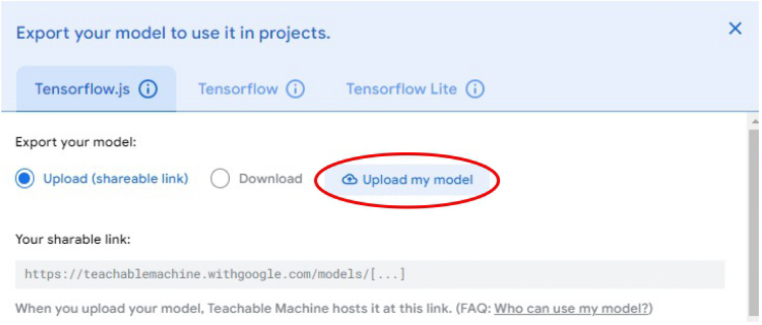
Step 15 - Operating instructions.

**Fig. 29:**
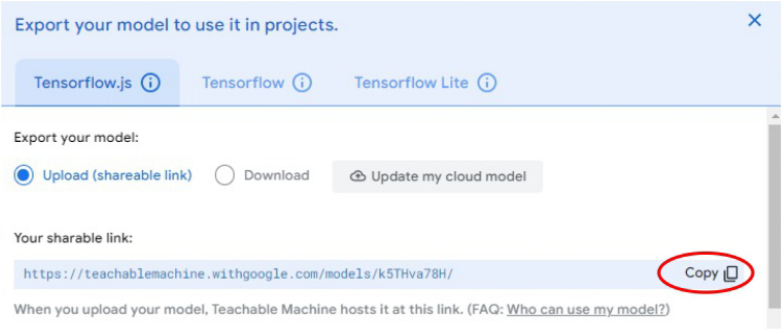
Step 16 - Operating instructions.

**Fig. 30:**
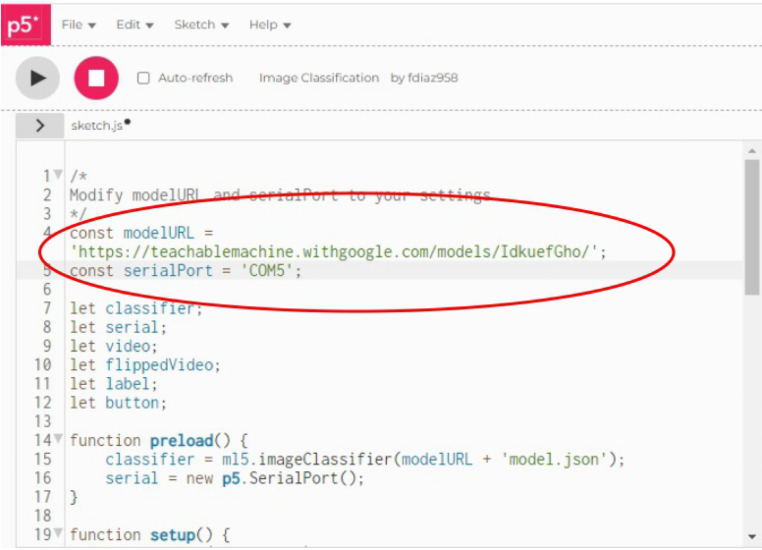
Step 17 - Operating instructions.

**Fig. 31:**
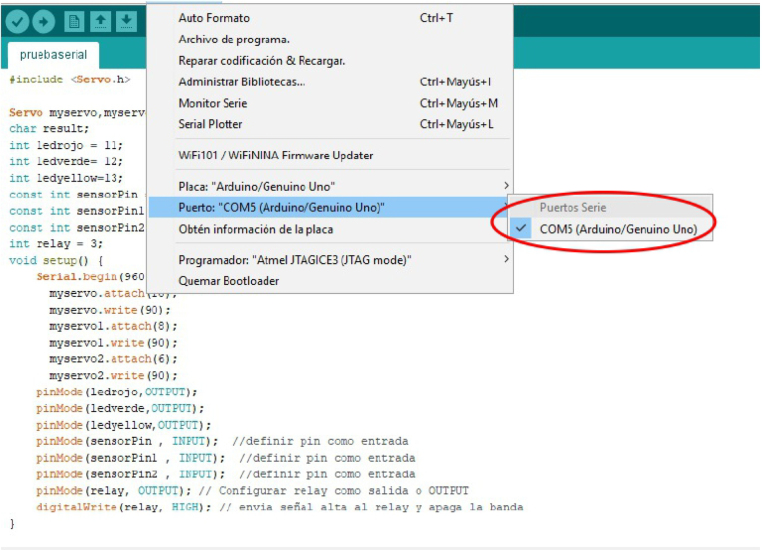
Step 18 - Operating instructions.

**Fig. 32:**
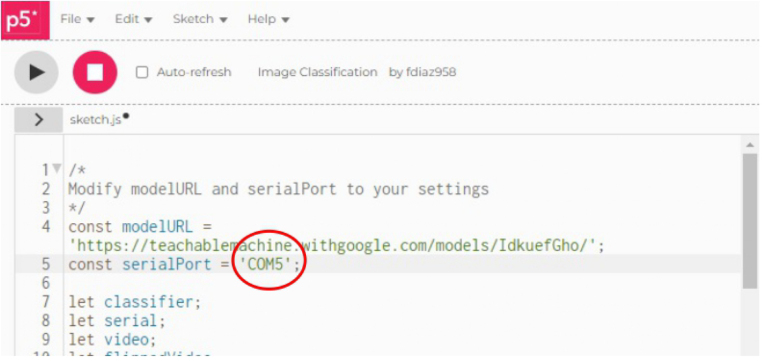
Step 19 - Operating instructions.

**Fig. 33:**
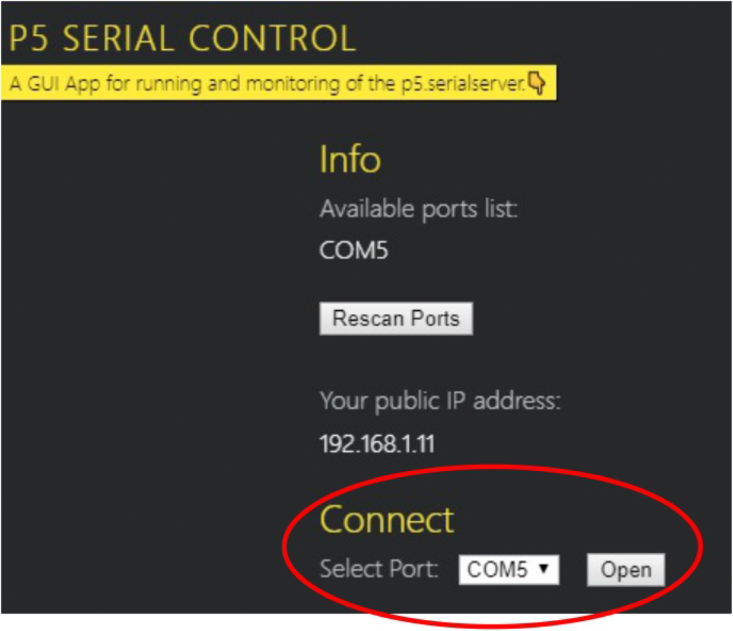
Step 20 - Operating instructions.

**Fig. 34:**
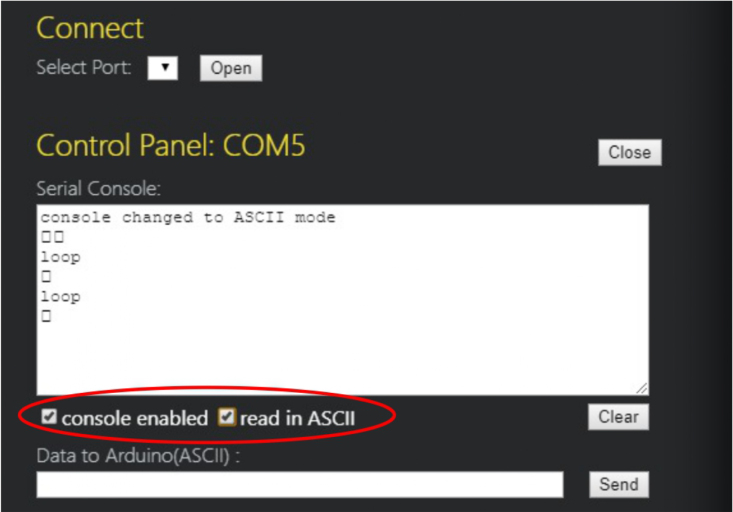
Step 21 - Operating instructions.

**Fig. 35:**
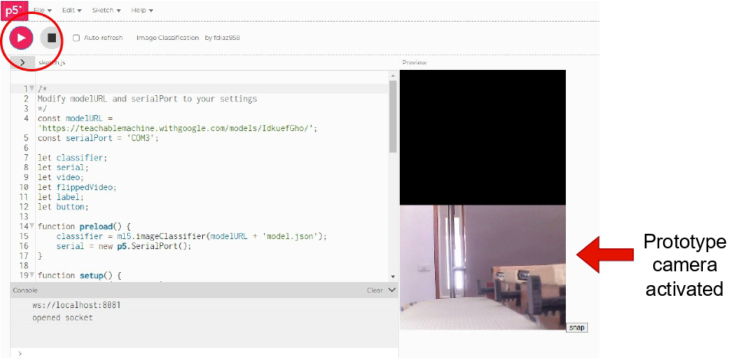
Step 22 - Operating instructions.

**Fig. 36:**
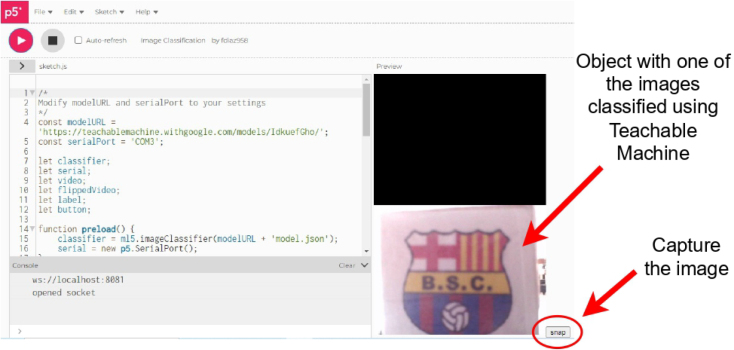
Step 23 - Operating instructions.

**Fig. 37:**
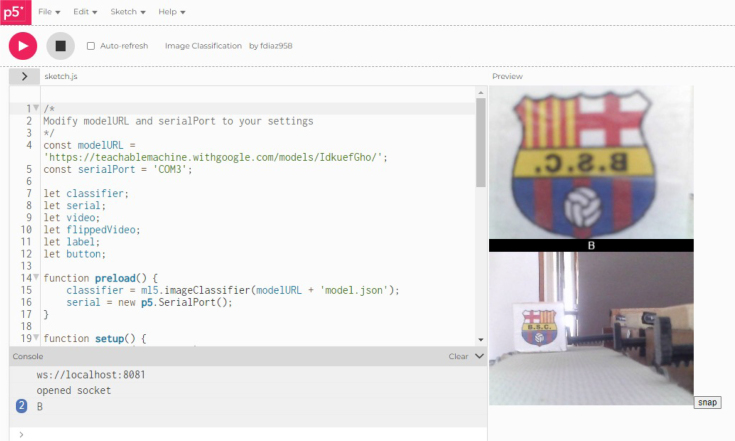
Step 24 - Operating instructions.

Listed below are the steps to follow for operating instructions:

1. Organize the system by making the physical connections between the computer and the prototype and verify that the prototype is connected to the electrical network. (See Fig. 18).
2. Verify that the prototype has the wooden blocks with the images for their respective classification.
3. Verify that the camera is connected to the PC on the conveyor belt.
4. Verify that the PC has Internet access.
5. Enter via the Internet to the link of the *teachable machine by Google* (https://teachablemachine.withgoogle.com/)
6. On the teachable machine by Google page click on the option “Get Started” (see Fig. 19).
7. In the New Project options that are displayed click on “Image Project”. (See Fig. 20)
8. In the window that appears click on the “Standard image model” option. This option will allow you to use the PC camera to capture the images and perform the training. (See Fig. 21)
  • In the window that appears, you must define the classes (Objects - wooden blocks with adhesive images) that will allow you to start the classification and training stage.
9. The names of the classes must be defined with a single character. (See Fig. 22)
  • Four classes will be used for this exercise: class A, class B, class C, and class D.
  • At the bottom of the classes, you have the option to create more classes and assign names to them using letters.
10. Then click on the class A webcam option to use the PC camera to capture the images of the wood blocks that correspond to that class. (See Fig. 23)
11. When the camera is turned on, place it in front of the wood block showing the image and click and hold where it says: “hold down to record”. (See Fig. 24)
  • Recommendation: move the block of wood so that it takes images from different angles. A minimum number of photos is 100 image samples.
  • Perform the same procedure with the other classes and the different blocks with their respective images.
12. Then click on the “prepare model” option until the “prepared model” information appears, and the preview option shows the classification output. (See Fig. 25)
13. To test the model, one of the images (wood blocks) of the three classes is placed in front of the camera and immediately the output will show the classification percentage of the image. (See Fig. 26)
14. Then, to simulate the scenario of the image classification system with respect to a real environment, we use the conveyor belt. To do this, click on the option “export model”. (Ver Fig. 27)
15. Then in the “upload my model” option of the tensorflow js tab click on “upload my model”. (See Fig. 28)
16. Once the model has been uploaded, it appears in the option “update my model” and we click on the option “copy”.
  • The model can be tested by opening it as an already defined system by checking the classification of the images used for training (see Fig. 29).
17. Then open the editor program p5.js, by clicking on the link: https://editor.p5js.org/fdiaz958/sketches/QyndWjeyH
  • In the window that appears, replace the link of the model we copied from the teachable machine with the model-URL that appears in the p5.js editor. (See Fig. 30)
18. For the communication between the pc and the electronic elements, an Arduino is used, when connecting it, it is verified in which port of the pc it is connected. (See Fig. 31) (For more information about the program see “pruebaserial.zip” at osf.io/4bdku in ‘Software’ folder.)
19. Place the port used by the Arduino in the p5.js code. (See Fig. 32)
  • In the same window that appears in the p5.js editor program, verify that the port described is the same port used by the Arduino.
20. Open the p5.serialcontrol program (a portable program that allows opening the ports to make connections from the Arduino to the serial code). (For more information about the program see “p5.serialcontrol-win32-x64.zip” at osf.io/4bdku in ‘Software’ folder.)
  • In the window that appears click on Rescan ports (rescan ports) and verify that the selected port is the same for the Arduino, then click on open (see Fig. 33).
21. In the same p5.serialcontrol window, select the console enabled, and read in ASCII options. (See Fig. 34)
  • This is to use the online code that sends the Arduino serial through the PC– Arduino—actuators and sensors.
22. Then, in the p5.js editor program that is open, click on the option shown with the play symbol (see the image on the left), and you will see on the right side that the prototype camera is activated. (See Fig. 35)
23. Then locate an object with one of the classified images and click on “snap” to capture the image (see Fig. 36).
24. When the image is captured, a signal will be sent to the Arduino, which will read the serial to activate the band, moving the object with the classified image until it is detected by the sensor located in the designated position for that class, the stop allows the band to stop, and at the same time, the servomotor pushes the wooden block with the image to the storage box (see Fig. 37).

## 7 Validation and characterization

***Technical profile of prototype***

The technical profile of the developed prototype is presented in Table 4.

***Validation***

**Table 4:** Technical profile of prototype.

Technical aspects	Description
Input voltage	120 V
Max. Current	0.25 A
Max. Power	30 W
Device weight	4 kg
Structure material	Medium Density Fiberboard (MDF), density 800 kg/m${}^{3}$, thickness 4 mm
Software	Teachable Machine/P5.js/p5.serialport

The validation of the functioning of the prototype can be observed in the link of this video https://www.youtube.com/watch?v=DczXmDnY30E

The prototype was validated through a process that involved expert judges in the field of artificial intelligence, as well as teaching activities with students using a didactic sequence designed specifically for this purpose.

The prototype’s ability to teach artificial intelligence concepts to high school students was evaluated by expert judges. The evaluation focused on aspects such as device usability, clarity of instructions, quality of proposed activities, and effectiveness of feedback.

On the other hand, teaching activities with students were conducted through a carefully designed didactic sequence that included pre-tests and posters to evaluate the impact of the prototype on student learning. The results of these activities showed that the prototype was helpful in improving students’ understanding of artificial intelligence concepts. It also increased their interest and motivation to learn about the topic.

The results obtained from student activities will be published elsewhere, with the goal of promoting the integration of this technological design into classroom setups.

***Capabilities***

• Recreate a practical scenario for object classification using images within a real-world application context.
• This device enables the recreation of other practical scenarios for object classification from images in a real-life application context.
• It is a device that is both portable and easy to assemble.
• It is a low power consumption device.
• It is simple to program as the interface is user-friendly and intuitive.
• The components of the device are of low cost.

***Limitations***

• The device has no backup or automatic recovery in the event of an interruption.
• The device has no visual indicators or alarms to inform the user of the operating status.
• This device only operates connected to the grid, so its modification for the use of alternative energies, such as photovoltaic, could be considered.
• The fact that the software is web-based means that it requires Internet access to function properly. This can be an obstacle in environments where connectivity is not constant or where it is preferred to work offline.

## CRediT authorship contribution statement

**Eduardo Orozco:** Writing – review & editing, Writing – original draft, Validation, Software, Resources, Methodology, Investigation, Funding acquisition, Formal analysis, Conceptualization. **Paulo C. Cárdenas:** Writing – review & editing, Supervision. **Jesús A. López:** Writing – review & editing, Supervision, Conceptualization. **Cinthia K. Rodriguez:** Writing – original draft, Visualization.

## Declaration of competing interest

The authors declare that they have no known competing financial interests or personal relationships that could have appeared to influence the work reported in this paper.
